# Biomarkers for diagnosis of neonatal infections: A systematic analysis of their potential as a point-of-care diagnostics

**Published:** 2011-12

**Authors:** Mahbuba Meem, Joyanta K. Modak, Roman Mortuza, Mahboob Morshed, Mohammad Shahidul Islam, Samir K. Saha

**Affiliations:** Child Health Research Foundation (CHRF), Department of Microbiology, Dhaka Shishu Hospital, Dhaka, Bangladesh

## Abstract

**Background:**

Neonatal infections annually claim lives of 1.4 million neonates worldwide. Until now, there is no ideal diagnostic test for detecting sepsis and thus management of possible sepsis cases often depends on clinical algorithm leading to empirical treatment. This often results in unnecessary antibiotic use, which may lead to emergence of antibiotic resistance. Biomarkers have shown great promise in diagnosis of sepsis and guiding appropriate treatment of neonates. In this study, we conducted a literature review of existing biomarkers to analyze their status for use as a point-of-care diagnostic in developing countries.

**Methods:**

PubMed and EMBASE database were searched with keywords, ‘infections’, ‘neonates’, and ‘biomarkers’ to retrieve potentially relevant papers from the period 1980 to 2010. Leading hospitals and manufacturers were communicated to inquire about the cost, laboratory requirements and current standing of biomarkers in clinical use.

**Results:**

The search returned 6407 papers on biomarkers; 65 were selected after applying inclusion and exclusion criteria. Among the studies, C-reactive protein (CRP), procalcitonin (PCT) and interleukin 6 (IL-6) were the most widely studied biomarkers and were considered to be most promising for diagnosing neonatal infections. About 90% of the studies were from developed countries; more than 50% were from Europe.

**Conclusions:**

Extensive work is being performed to find the diagnostic and prognostic value of biomarkers. However, the methodologies and study design are highly variable. Despite numerous research papers on biomarkers, their use in clinical setting is limited to CRP. The methods for detection of biomarkers are far too advanced to be used at the community level where most of the babies are dying. It is important that a harmonized multi-site study is initiated to find a battery of biomarkers for diagnosis of neonatal infections.

Most developing countries have witnessed substantial declines in mortality among children <5 years of age (1,2). In contrast, neonatal mortality has remained relatively constant, with an estimated 3.6 million annual neonatal deaths globally (2-5). Neonatal mortality now accounts for about 40%-50% of under-five child deaths (4-6). More than 90% of these deaths occur in the poorest countries of Asia and Africa (7). Suspected infections, including sepsis, pneumonia and meningitis (hereafter referred to as “infections”) account for an estimated 1.4 million neonatal deaths worldwide every year (5,6).

Low and middle income countries are trying different modalities to achieve MDG4 by 2015. The common intervention is community-based diagnosis of possible sepsis cases, using clinical algorithms and treatments with empirical antibiotics. Highly sensitive algorithms based therapies have performed well in reducing child mortality, irrespective of the antibiotic therapy used (6,8). However, blood culture, as the gold standard for diagnosis, from these algorithm-positive cases yielded bacterial isolates only in 5%-10% of cases. This jeopardized the credibility of the “gold” standard. In recent years, with the advancement of these techniques like real time polymerise chain reaction (RT-PCR) for specific genome and broad range targets, the use of molecular approaches has become common for aetiological diagnosis (9). Although a recent meta-analysis showed that the molecular tests cannot increase the detection frequency of aetiology more than what blood culture already captures (9). Hence it is becoming increasingly important to find a tool to differentiate sick newborns with or without infection, especially to minimize the indiscriminate use of antibiotics. In the last few years, biomarkers, triggered by the host immune system in response to infections, have been targeted as potential indicator for diagnostic and prognostic purposes.

This study was taken up to conduct a structured literature overview on the existing biomarkers for diagnosis of neonatal infections/sepsis and to elucidate their relative potential to be used in resource-poor settings. In addition, the study also investigated the instrumental requirements for detection of biomarkers and the extent of their use in clinical practice.

## METHODS

### Selection of biomarkers for analysis

After a preliminary examination of the available literature, we consolidated the list of biomarkers for further review. These markers were selected based on the number of papers published on the topic and their potential to be used for diagnosis and prognosis of neonatal infection. Biomarkers included in this analysis are as follows:

*Acute phase proteins:* C– reactive protein (CRP), procalcitonin (PCT);

*Cytokines:* interleukin 6 (IL-6), interleukin 8 (IL-8), interferon – gamma (IFN-γ), tumor necrosis factor – alpha (TNF-α);

*Cell surface antigens:* CD 64, soluble intercellular adhesion molecule (sICAM).

### Search strategies

In order to carry out a landscape analysis to identify studies on the diagnostic performance of the aforementioned biomarkers, we searched PubMed and EMBASE bibliography databases. Search strategies for both databases were carefully built to maximize the sensitivity of our search. A combination of text words and subject heading terms specific to each database (MeSH terms for PubMed and EMTREE terms for EMBASE) were used to develop the search strategy (**Table 1**).

**Table 1:** Search strategy, restricted to age (newborn), subject (humans) and time period (January 1980 to April 2010)

EMBASE:	('newborn'/exp OR newborn OR 'newborn'/syn OR 'newborns':ab,ti OR 'neonates':ab,ti OR 'infants':ab,ti) AND ('infection'/exp OR infection OR 'infection'/syn OR 'infections' OR 'sepsis'/exp OR sepsis OR 'sepsis'/syn OR 'bacterial infection'/exp OR 'bacterial infection' OR 'infections':ab,ti OR 'bacterial infection'/syn OR 'bacteremia'/exp OR bacteremia OR 'bacteremia'/syn OR 'septicemia'/exp OR septicemia OR 'septicemia'/syn OR 'systemic inflammatory response syndrome'/syn OR 'systemic inflammatory response syndrome'/exp OR 'systemic inflammatory response syndrome' OR 'meningitis'/exp OR 'meningitis' OR 'meningitis'/syn) AND ('c reactive protein'/exp OR 'c reactive protein' OR 'c reactive protein'/syn OR 'procalcitonin'/exp OR procalcitonin OR 'pct':ab,ti OR 'tumor necrosis factor alpha'/exp OR 'tumor necrosis factor alpha' OR 'tumor necrosis factor alpha'/syn OR 'tnf alpha' OR 'tnf-alpha':ab,ti OR 'gamma interferon'/exp OR 'gamma interferon' OR 'gamma interferon/syn' OR 'ifn-gamma':ab,ti OR 'ifn gamma':ab,ti OR 'intercellular adhesion molecule 1'/exp OR 'intercellular adhesion molecule 1' OR 'intercellular adhesion molecule 1'/syn OR 'icam 1':ab,ti OR 'cd64 antigen'/exp OR 'cd64 antigen' OR 'cd64 antigen'/syn OR 'cd64':ab,ti OR 'interleukin 6'/exp OR 'interleukin 6' OR 'interleukin 6'/syn OR 'il 6':ab,ti OR 'il-6':ab,ti OR 'interleukin 8'/exp OR 'interleukin 8' OR 'interleukin 8'/syn OR 'il 8':ab,ti OR 'il-8':ab,ti) AND ('diagnosis'/exp OR diagnosis OR 'diagnosis'/syn OR 'biological marker'/exp OR 'biological marker' OR 'biological marker'/syn OR markers:ab,ti OR biomarkers:ab,ti OR test:ab,ti OR tests:ab,ti OR indicators:ab,ti)
PubMed/Medline:	(neonat* [tw] OR newborn [mh] OR newborn [tw] OR newborns [tw] OR neonate [tw] OR neonates [tw] OR baby [tw] OR babies [tw] OR infant [tw] OR Infants [tw]) AND (“sepsis” [mh] OR sepsis [tw] OR “bacterial Infections” [mh] OR (septic [tw] AND shock [tw]) OR “systemic inflammatory response syndrome” [mh] OR “systemic inflammatory response syndrome” [tw] OR infection [tw] OR infections [tw] OR bacteremia [tw] OR bacteraemia [tw] OR bacteremias [tw] OR bacteraemias [tw] OR septicemia [tw] OR septicemias [tw] OR septicaemia [tw] OR septicaemias [tw] OR bacteremic [tw] OR bacteraemic [tw] OR bacterial [tw] OR viremia [tw] OR viremias [tw] OR viraemia [tw] OR viraemias [tw] OR Viremic [tw] OR viraemic [tw] OR fungemic [tw] OR fungemia [tw] OR fungemias [tw]) AND ((“Diagnosis” [mh] AND (markers [tw] OR marker [tw])) OR markers [tw] OR marker [tw] OR “biological markers” [mh] OR biomarker [tw] OR biomarkers [tw] OR (“sensitivity and specifity” [mh] AND (sensitivity [tw] OR specificity[tw])))

The search strategy also adapted individual biomarker specific final queries and ran the search to ensure retrieval of maximum papers. A total of 4868 citations from PubMed and 1539 citations from EMBASE were retrieved. These references were imported into separate libraries using the EndNote software (Thomson Reuters, Philadelphia, PA, USA). The libraries were later merged, and the duplicates were removed. Two reviewers independently screened the titles and abstracts of the retrieved citations to find the articles that were deemed relevant.

### Inclusion criteria

For inclusion, the abstract and titles were screened based on the following predetermined criteria: i) the subject population is newborns, ii) the subjects are with culture proved sepsis or suspected infection based on clinical algorithm and iii) the article evaluated any of the proposed biomarkers for diagnosis and/or prognosis of neonatal infections.

The exhaustive search based on the titles and abstracts returned a broad spectrum of infection related studies from which only the cases of sepsis, urinary tract infection, meningitis, pneumonia, respiratory tract infections and umbilical cord infections were considered.

Finally, full text articles with following criteria were included for analysis: i) the age of the newborns ranged from 0 days to 59 days and ii) diagnostic performance of target biomarkers are explored in clinical and/or in culture-confirmed cases of sepsis.

### Exclusion criteria

It was challenging to select the relevant articles for this analysis from the large number of papers retrieved (n=6407) based on the above mentioned selection criteria. To make a comprehensive list of appropriate papers, we excluded the articles that dealt with malaria, HIV infection, hepatitis, toxoplasmosis, gestational diabetes, bronchopulmonary dysplasia, antenal and maternofetal studies, in-vitro studies, transplant immunology studies, polymorphisms, necrotizing enterocolitis, foreign languages other than English, letters, comments and editorials and other non-research publication types.

### Data extraction

Available full papers were downloaded from PubMed, EMBASE and HINARI sources. Requests for reprints were sent to the authors of the papers which were not available from these sources. Data were extracted and compiled in Excel spreadsheet with the following column headings: Name of Biomarker, Study Title, First Author, Year, Setting, Country, Clinical Characteristics, Sample Size, Age, Specimen source, Method, Cut off, Cost, Sensitivity, Specificity, Positive Predictive Value (PPV), and Negative Predictive Value (NPV).

For a point of reference, we contacted the leading hospitals of several developed and developing countries to learn what tests/biomarkers are currently being used at their clinical settings. We also contacted major diagnostics manufacturers to inquire about the direct costs and laboratory requirements for assaying each of the biomarkers.

## RESULTS

**Figure 1:**
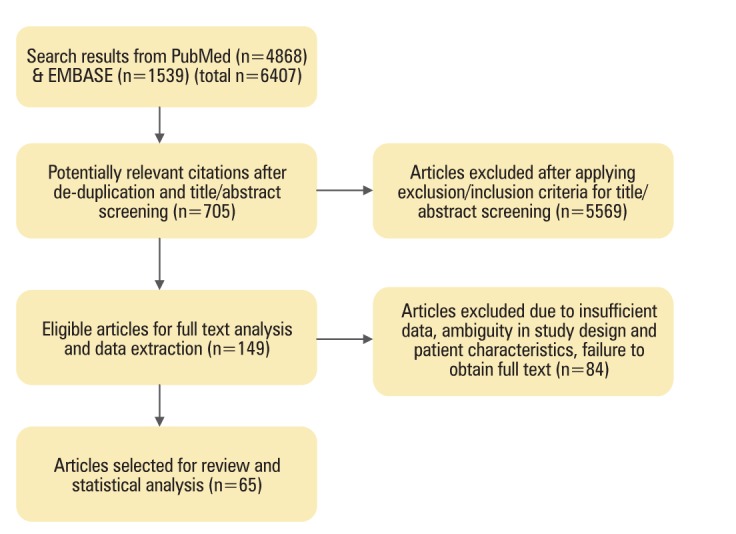
Search strategy and identified articles.

Out of 705 potentially relevant papers, 65 were selected for final review after exclusion of 640 papers for the lack of sufficient information, ambiguity in study design and patient characteristics, failure to obtain full-text article or absence of other inclusion criteria (**Figure 1**).

### Prevalence of research on biomarkers

**Figure 2:**
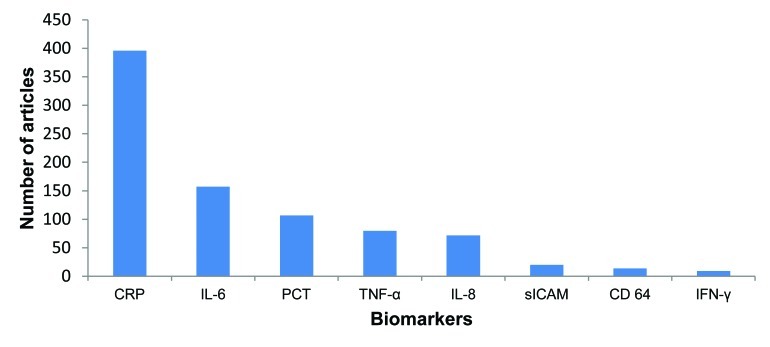
Distribution of studies according to biomarkers studies. CRP – C-reactive protein, IL – interleukin, TNF – tumor necrosis factor, sICAM (soluble intercellular adhesion molecule, IFN – interferon.

Review of relevant papers, published during January1980 to April 2010, revealed that CRP was the most extensively studied biomarker (n=396), followed by IL-6 (n=157), PCT (n=107), TNF-alpha (n=80), IL-8 (n=72), sICAM (n=20), CD64 (n=14), IFN-γ (n=9) **(****Figure 2****).** However, even the less frequent markers showed promise in respect of their ability to differentiate the sepsis from non-sepsis cases.

### Heterogeneity of the studies

Biomarker research studies widely differed by study groups in respect to their inclusion criteria for patients, case definition, test methodologies and cut off values for markers (**Table 2**). The range of cut off value was as wide as 0.2 to 95 mg/mL for CRP, 0.34 to 100 ng/mL for PCT, 3.6 to 500 pg/mL for IL-6; and 1 to 1000 pg/mL for IL-8. Accordingly, sensitivity and specificity of the tests also varied widely among the studies. For convenience of interpretation of sensitivity and specificity, we divided the studies in smaller subgroups based on their cut off values used by the study groups (**Table 2**).

**Table 2:** Sensitivity, specificity and cut-off values of biomarkers in reviewed studies*

Biomarker	Cut-off range	Cut-off sub-ranges	Percentage of papers	Sensitivity ranges (%)	Specificity ranges (%)	Cut-off	Sensitivity	Specificity
**CRP**	0.2–95 mg/L	0.2–10 11–30 31–95	70 15 15	41–96 33–56 23–87	72–100 74–96 48–98	mean=17.1 median=10	mean=66.53 median=69 (IQR=26.3)	mean=86.14 median=90 (IQR=13.9)
**PCT**	0.34 –100 ng/mL	0.34–1.0 2–10 11–100	48 39 13	58–100 59–95 21–95	50–100 50–100 87–100	mean=8.92 median=1.17	mean=77.93 median=8 (IQR=14.8)	mean=81.84 median=82.5 (IQR=16.6)
**IL-6**	3.6–500 pg/mL	2–10 11–30 31–100 101–500	15 34 31 20	88–96 61–90 57–100 74–97	66–89 56–90 43–100 70–100	mean=76.49 median=30	mean=77.87 median=80 (IQR=29.9)	mean=78.61 median=78 (IQR=18.9)
**IL-8**	1–1000 pg/mL	0.6–100 101–1000	74 26	34–92 36–92	52–96 65–96	mean=220.53 median=70	mean=72.48 median=80 (IQR=15.5)	mean=80.57 median=82 (IQR=21.7)
**CD64**	different units used	–	–	79–100	81–96.8	not analyzable	mean=82.42 median=92 (IQR=17)	mean=82.79 median=88 (IQR=15)
**sICAM**	250–300 µg/L	–	–	78–80	61–90	mean=275 median=275	mean=79 median=79 (IQR=1)	mean=75.5 median=75.5 (IQR=14.5)
**TNF-α**	1.7–70 pg/mL	–	–	54–100	43–96.6	mean=18.94 median=7.5	mean=78.72 median=80.4 (IQR=22.7)	mean=81.4 median=93 (IQR=14.9)

CRP – C-reactive protein, IL – interleukin, TNF – tumor necrosis factor, sICAM (soluble intercellular adhesion molecule, IFN – interferon, IQR – interquartile range.

*Reported in refs 13-77.

### Characteristics of biomarkers

The mean cut-off point of CRP was 17 mg/L, with 66% sensitivity and 86% specificity. PCT appeared to be a more relevant marker than CRP for diagnosing bacterial sepsis at earlier stages, with the mean sensitivity of 77.93%, specificity of 81.84%, and a cut-off of 8.92 ng/mL. For IL-6, the mean sensitivity at “zero hour” was 77.87%, specificity was 78.61%, and the mean cut- off value was 76.49 pg/mL (**Table 2**). The mean value of cut-off for IL-8 was 220.53 pg/mL, sensitivity 72.48% and specificity 80.57% (**Table 2**).

CRP and PCT have been extensively studied and compared for their efficacy to diagnose sepsis cases in young infants. Overall, the studies reported that the optimum sensitivity and specificity for CRP was obtained during the window of 24-48 hours after the onset of symptoms. On the other hand, PCT was sensitive enough to detect the cases much earlier than CRP. However, some studies also suggested that serial measurements of CRP over a period of 2-3 days after onset clinical symptom, using varying cut-off values, improved the diagnostic performance of CRP (10,11).

CD64 demonstrated the mean sensitivity of 92% and a specificity of 82.79% during the first 24 hours of infection. sICAM yielded the mean sensitivity of 79% and specificity of 75.5%. Finally, the mean sensitivity of TNF-α was 78.72%, and the specificity was 81.4% (**Table 2**). Unfortunately, we could not find any analyzable data for IFN-γ from any of the relevant studies.

### Minimum laboratory requirements and cost analysis

According to retrieved studies, the major techniques for detecting biomarkers were immunoassays, and cell sorting for CD64. The immunoassays were usually accompanied by a variety of readers to quantify the level of specific markers. Most of the studies used enzyme-linked immunosorbent assay (ELISA) readers for quantification along with other tests like immunoturbidimetric and nephelometric assays. These methods, except ELISA, were rarely available at the resource-poor settings.

The detection method for CRP as the extensively studied and widely used marker was also immunoassay, usually using an ELISA reader. In resource-poor settings, qualitative or semiquantitative latex agglutination test is also used for the detection of CRP. Other immunoassays are only available at the tertiary level and/or private commercial facilities in low income countries. Some studies used immunoluminometry and chemiluminescence for the detection of PCT. Immunochromatographic tests (ICT) was also reported for PCT in a few studies, but these were still at the developmental stage and only at research level (12). Flow cytometry was invariably used by all the studies for detection of cell surface antigen CD64 and the literature revealed very little information about the cost associated with these techniques (13-77).

### Global distribution of biomarker research

**Figure 3:**
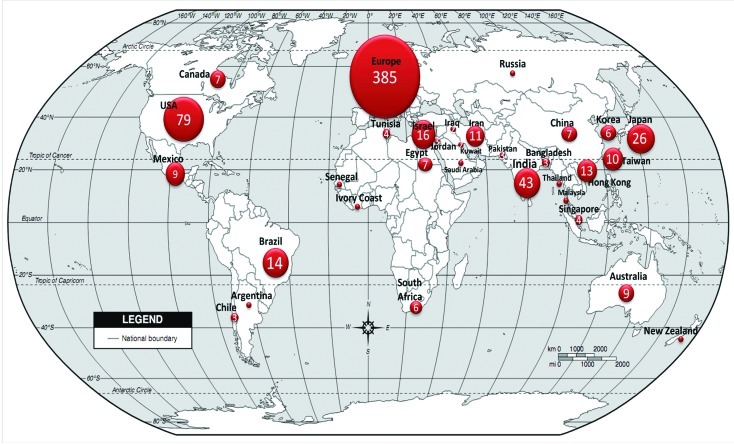
Geographical distribution of published research on biomarkers in the last three decades (January 1980 to April 2010).

We tried to establish the geographical distribution of biomarker research based on retrieved studies. Research on biomarkers was mostly confined to the developed countries with a large share of the total numbers of articles (385/705, 55%) from Europe, followed by North America (95/705, 13%). In contrast, only 9.5% (68/705) papers were from South Asia and Africa (**Figure 3**); the regions where more than a third of neonatal deaths occur.

## DISCUSSION

Biomarkers may have great potentials for diagnosis of neonatal sepsis and have been studied for more than two decades. The frontiers of research in biomarkers as diagnostic tools for detecting neonatal sepsis have progressed considerably over the years and persist in advancing novel technologies. This review suggests that many newer biomarkers have come into play, and thorough investigations on them are in progress.

Among the numerous biomarkers in the field of neonatal sepsis diagnosis, this review identified 8 predominant markers, as determined by number of publications: CRP, PCT, IL-6, IL-8, IFN-γ, TNF-α, CD64 and sICAM. Of these, CRP was the most widely used diagnostic and prognostic marker. Despite its limitation due to late appearance and persistence for relatively longer period (76), it was used as the standard marker to measure the potential and efficacy of newer biomarkers. Among other markers, PCT has come up as more promising, with the comparative advantage of early detection in sepsis and quick reduction in its levels in response to appropriate therapy (72). PCT also has the additional advantage of being specifically responsive to bacterial infections and not viral (73). On the other hand, IFN-γ seems to be particularly responsive to viral infection at very early stage of infection (74).

Based on the available data about the detection time, the markers could be classified into three groups; early phase (IL-6, IL-8, CD64, sICAM, TNF-α and IFN-γ), mid phase (PCT) and late phase (CRP). The unique dynamics of appearance and disappearance of specific markers would be useful for possible multiplexing to capture the neonatal infection cases irrespective of their disease status.

Other biomarkers have also showed promise, and most of them revealed potential for detection of sepsis at very early stage of the disease. IL-6 demonstrated a high potential with the ability to detect the cases at very early stage of infection and monitor the appropriateness of therapy, based on its characteristic early appearance and short half life (75). Newer markers like sICAM and CD64 also have the potential to detect sepsis cases at very early stage of disease, with high sensitivity but compromised specificity (57-62).

With all possible and definite potentials of biomarkers, none of them is currently in use for patient care, except CRP. The review identified several reasons for this slow transition of biomarkers from the research laboratories to their real-life use in clinical care. The main cause of this hindrance is the heterogeneity between the research protocols used by different groups. The study designs are heterogenic with respect to cut off values used to define positivity, which sometimes varied by about 100 folds (**Table 2**). In some studies different threshold levels were used for same biomarker, based on the duration of illness at the time of collection of blood (10,11). This is an impractical approach to be implemented at any clinical setting.

Defining “zero hour” is an important parameter to characterize the biomarkers as early or late infection detectors. However, this definition varied from study to study as it was mostly decided based on the blood collection time, and only few studies considered the first onset of illness. The requirement for early onset diagnosis is not usually relevant for low and middle income countries where care seeking behaviour is poor, and thus the babies are brought to the hospital when the disease process has already progressed to a severe state.

We also observed that case definition for sepsis differed from study to study. In majority of the studies, sepsis was defined based on clinical algorithm, which also varied from study to study. Furthermore, there were studies where only culture-proved cases were considered as sepsis. This is a challenging issue: if we consider all clinically suspected sepsis cases as true, we run the risk of diluting true sepsis cases; if not, then we are possibly missing out the actual infections which are not captured by blood culture.

Additionally, >90% of biomarker studies were from developed countries, and >50% were specifically from Europe. Therefore, there is almost no data from developing countries where the populations are different with respect to their exposure to microbes, aetiology of infection, nutritional status, time for care-seeking behaviour, and other factors.

In conclusion, biomarker research has many limitations, its progress has slowed down and research results are far from reaching the population where biomarkers are needed most.

Several steps are needed to facilitate the uptake of biomarkers as tools to diagnose neonatal infections in the developing countries; i) a multi-country and multi-site study using a harmonized protocol to detect the most promising biomarkers, ii) formulation of their use in single and/or multiplex format, iii) development of point care device and their trial in the facility level and iv) validation of point of care device in large population based sites of multiple countries.
